# Anthocyanin and Carotenoid Contents in Different Cultivars of Chrysanthemum (*Dendranthema grandiflorum* Ramat.) Flower

**DOI:** 10.3390/molecules200611090

**Published:** 2015-06-15

**Authors:** Chang Ha Park, Soo Cheon Chae, Soo-Yun Park, Jae Kwang Kim, Yong Joo Kim, Sun Ok Chung, Mariadhas Valan Arasu, Naif Abdullah Al-Dhabi, Sang Un Park

**Affiliations:** 1 Department of Crop Science, Chungnam National University, 99 Daehak-ro, Yuseong-gu, Daejeon 305-764, Korea; E-Mail: parkch804@gmail.com; 2 Department of Horticultural Science, College of Industrial Sciences, Kongju National University, Daehoe-ri, Yesan-kun, Chungnam 340-720, Korea; E-Mail: scchae@kongju.ac.kr; 3 National Academy of Agricultural Science, Rural Development Administration, Wanju-gun, Jeollabuk-do 565-851, Korea; E-Mail: psy22@korea.kr; 4 Division of Life Sciences and Bio-Resource and Environmental Center, Incheon National University, Yeonsu-gu, Incheon 406-772, Korea; E-Mail: kjkpj@inu.ac.kr; 5 Department of Biosystems Machinery Engineering, Chungnam National University, 99 Daehak-ro, Yuseong-gu, Daejeon 305-764, Korea; E-Mails: babina@cnu.ac.kr (Y.J.K.); sochung@cnu.ac.kr (S.O.C.); 6 Department of Botany and Microbiology, Addiriyah Chair for Environmental Studies, College of Science, King Saud University, P. O. Box 2455, Riyadh 11451, Saudi Arabia; E-Mail: mvalanarasu@gmail.com; 7 Visiting Professor Program (VPP), King Saud University, P.O. Box 2455, Riyadh 11451, Saudi Arabia

**Keywords:** chrysanthemum, *Dendranthema grandiflorum*, cyanidin 3-glucoside, cyanidin 3-(3ʺ-malonoyl) glucoside, lutein, zeaxanthin, β-cryptoxanthin, α-carotene, 13-*cis*-β-carotene, *trans*-β-carotene, 9-*cis*-β-carotene

## Abstract

The flowers of twenty-three cultivars of *Dendranthema grandiflorum* Ramat. were investigated to determine anthocyanin and carotenoid levels and to confirm the effects of the pigments on the flower colors using high-performance liquid chromatography (HPLC) and electrospray ionization-mass spectrometry (ESI-MS). The cultivars contained the anthocyanins cyanidin 3-glucoside (C3g) and cyanidin 3-(3ʺ-malonoyl) glucoside (C3mg) and the following carotenoids: lutein, zeaxanthin, β-cryptoxanthin, 13-*cis*-β-carotene, α-carotene, *trans*-β-carotene, and 9-*cis*-β-carotene. The cultivar “Magic” showed the greatest accumulation of total and individual anthocyanins, including C3g and C3gm. On the other hand, the highest level of lutein and zeaxanthin was noted in the cultivar “Il Weol”. The cultivar “Anastasia” contained the highest amount of carotenoids such as *trans*-β-carotene, 9-*cis*-β-carotene, and 13-*cis*-β-carotene. The highest accumulation of β-cryptoxanthin and α-carotene was noted in the cultivar “Anastasia” and “Il Weol”. Our results suggested that ‘Magic”, “Angel” and “Relance’ had high amounts of anthocyanins and showed a wide range of red and purple colors in their petals, whereas “Il Weol’, “Popcorn Ball’ and “Anastasia” produced higher carotenoid contents and displayed yellow or green petal colors. Interestingly, “Green Pang Pang”, which contained a high level of anthocyanins and a medium level of carotenoids, showed the deep green colored petals. “Kastelli”, had high level of carotenoids as well as a medium level of anthocyanins and showed orange and red colored petals. It was concluded that each pigment is responsible for the petal’s colors and the compositions of the pigments affect their flower colors and that the cultivars could be a good source for pharmaceutical, floriculture, and pigment industries.

## 1. Introduction

The garden chrysanthemum, *Dendranthema grandiflorum* Ramat. (*Chrysanthemum morifolium* Ramat., Kitamura, 1978), is a perennial plant belonging to the *Asteraceae* (Compositae) family. It has been cultivated for more than 3000 years. The flowers of *D. grandiflorum* have been consumed as herbal medicines, beverages, and vegetables in China, Japan, Thailand, and the Republic of Korea for centuries [1,2,3]. They are also among the most popular cut flowers in the floriculture industry of China, United States, and Europe due to their high ornamental features, including various floral colors and shapes, uniform flowering, and many spray flowers [4,5].

Flower color depends on the grade of accumulation of secondary compounds, including flavonoids, carotenoids, or betalains. Anthocyanins, members of the flavonoid group of phytochemicals, are mainly involved in color development of a wide range of orange to red and purple to blue flowers. Flower colors play a major role in attracting pollinators, protecting against damage from ultraviolet irradiation, and as key signals between plants and microbes [6,7,8]. Chrysanthemum cultivars are considered a good source for the extraction of anthocyanins since these cultivars have numerous flowers in a single plant with wide variations of flower color [8].

Carotenoids are natural pigments that impart yellow, red, or orange colors to flowers and are constituted by C_40_ isoprenoid compounds with or without epoxy, hydroxy, and keto groups [9]. Most plants have similar carotenoid contents, including both β,ε-carotenoids and β,β-carotenoids, in their green tissues [10]. In contrast, the carotenoid profiles in non-green tissues show qualitative differences depending on plant species. For example, xanthophylls, which impart pale to deep yellow colors, accumulate significantly in the flower petals in most plants [6]. However, the petals of several plants such as *Calendula officinalis* are able to synthesize unique carotenoids such as lycopene that are absent in yellow petals and present in orange to red petals [11]. These distinctive carotenoid compositions enable plants to have various petal colors.

Pharmacological studies reported that chrysanthemum species contain secondary compounds having various biological characteristics. Various flavonoids, alkaloids, phenolic compounds, and triterpene constituents were identified from the aqueous methanol extract of a chrysanthemum flower. These components were found to have biological activities such as anti-inflammatory, antibacterial, antifungal, anti-spirochetal, anti-human immunodeficiency virus, and anti-oxidant activities [12,13,14].

Chrysanthemum cultivars have been studied in many scientific fields, including pharmacology, morphology, genetics, genetic engineering, and horticulture; however, few studies have investigated the biosynthesis of anthocyanins. Recently, It was reported that the total anthocyanin content in flower tepals of fifteen spray chrysanthemum cultivars grown at a polyhouse and under open field conditions [8]. 

The aim of this study is to determine anthocyanin and carotenoid contents responsible for petals colors in the twenty-three chrysanthemum cultivars and to confirm the effect of the pigments on the flower colors by using high-performance liquid chromatography (HPLC) and electrospray ionization-mass spectrometry (ESI-MS).

## 2. Results and Discussion

### 2.1. Analysis of Individual Anthocyanin Contents

**Figure 1:**
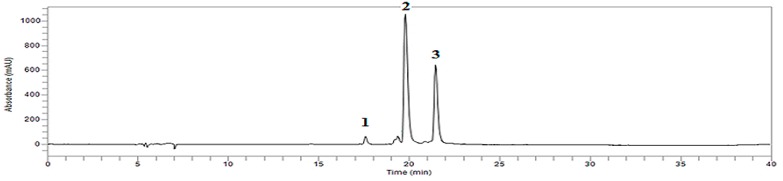
HPLC chromatogram of an anthocyanin profile of *Dendranthema grandiflorum* Ramat. Peak Identification: 1, Cyanidin 3-glucoside; 2, Cyanidin 3-(3ʹʹ-malonoyl)glucoside; 3, Unknown compound. Refer to Table 1 for the identification of each numbered peak.

The flowers of the twenty-three cultivars were analyzed for the biosynthesis of anthocyanins using HPLC and ESI-MS. The anthocyanin components were identified by their retention time, elution order, and fragmentation patterns. Five anthocyanin standards (malvidin-3-*O*-glucoside chloride: retention time (RT) 17.557; cyanidin-3-*O*-glucoside chloride: 17.701; pelargonidin-3-*O*-glucoside chloride: 18.925; peonidin-3-*O*-glucoside chloride: 19.751; and cyanidin-3-*O*-rutinoside chloride: 20.331), cyanidin 3-glucoside (C3g), cyanidin 3-(3ʺmalonoyl) glucoside (C3mg) were used for the anthocyanin analysis in the cultivars. The chromatogram showed three peaks corresponding to three different anthocyanins such as C3g, C3mg, and an unknown anthocyanin by the retention time (Figure 1). Table 1 indicates that peak 1 was shown to be a C3g ([M + H]^+^, *m*/*z* 449; MS/MS, *m*/*z* 277) and peak 2 corresponded to C3gm ([M + H]^+^, *m*/*z* 535; MS/MS, *m*/*z* 277). Peak 3 showed the presence of an unidentified type of cyanidin ([M + H]^+^, *m*/*z* 662; MS/MS, *m*/*z* 277).

Table 2 presents the significantly wide variation of individual anthocyanin contents, including C3g, C3mg, and unknown anthocyanin contents. The extract of “Magic’ showed the highest level of C3g and C3gm. Interestingly, the unknown anthocyanin was quantified in several cultivars. The cultivar “Relance” showed the significantly highest accumulation of the unknown anthocyanin, followed by “Angel”, “Magic”, and “Green Pang Pang”. The other cultivars also contained some amount of the unknown anthocyanin (Table 2). 

**Table 1:** Identification of anthocyanins from *Dendranthema grandiflorum* Ramat.

HPLC Peak No.	Anthocyanin	RT (min)	[M + H]^+^ (*m/z*)	MS/MS (*m/z*)
1	Cyanidin 3-glucoside	17.60	449	287
2	Cyanidin 3-(3ʹʹ-malonoyl)glucoside	19.33	535	287
3	Unknown compound	21.42	622	287

**Table 2:** Anthocyanin contents of the twenty-three *Dendranthema grandiflorum* Ramat. cultivars.

Cultivars	Anthocyanins (mg/g dry wt.)		
	C3g	C3mg	Unknown
Ford	ND g	ND j	ND i
Fire Pink	0.29 ± 0.15 d–g	3.54 ± 0.09 f	2.51 ± 0.30 f
Dark Pang Pang	0.26 ± 0.00 e–g	2.37 ± 0.17 g	3.60 ± 0.39 d
Borami	0.11 ± 0.06 fg	1.04 ± 0.08 i	0.93 ± 0.07 h
Yes Nuri	0.04 ± 0.06 g	0.25 ± 0.23 j	0.17 ± 0.06 i
Purple Ball	ND g	ND j	ND i
Magic	1.06 ± 0.54 a	11.32 ± 0.38 a	5.62 ± 0.16 c
Kastelli	0.45 ± 0.21 b–e	4.96 ± 0.05 d	2.55 ± 0.23 f
Green Pang Pang	0.72 ± 0.26 b	6.73 ± 0.11 b	3.92 ± 0.33 d
Yes Together	ND g	ND j	ND i
Mujigae	0.39 ± 0.19 c–f	4.07 ± 0.04 e	3.10 ± 0.39 e
Relance	0.53 ± 0.27 b–d	5.50 ± 0.45 c	6.97 ± 0.45 a
Anastasia	ND g	ND j	ND i
Gold King	ND g	ND j	ND i
Burning Ball	0.14 ± 0.07 e–g	1.49 ± 0.05 h	1.74 ± 0.17 g
Popcorn Ball	ND g	ND j	ND i
Fancy Ball	0.20 ± 0.09 e–g	2.19 ± 0.08 g	2.10 ± 0.15 g
Sodam Ball	ND g	ND j	ND i
Geumbangwul	ND g	ND j	ND i
Angel	0.66 ± 0.32 bc	6.69 ± 0.25 b	6.51 ± 0.62 b
Yes Song	0.21 ± 0.10 e–g	2.14 ± 0.02 g	1.27 ± 0.12 h
Il Weol	0.04 ± 0.01 g	0.27 ± 0.01 j	0.16 ± 0.02 i
Hwiparam	ND g	ND j	ND i

All values are written as the mean (mg/g) ± standard deviation (SD) of three replications. The amounts are expressed as mg of the target compound per g of the plant on a dry weight basis. Means values in a common letter were not significantly different at *p* < 0.05 using Duncan Multiple Range Test. The limit of detection was 0.05 mg/g dry wt. The background color in each column presents the flower color of the cultivar.

**Figure 2:**
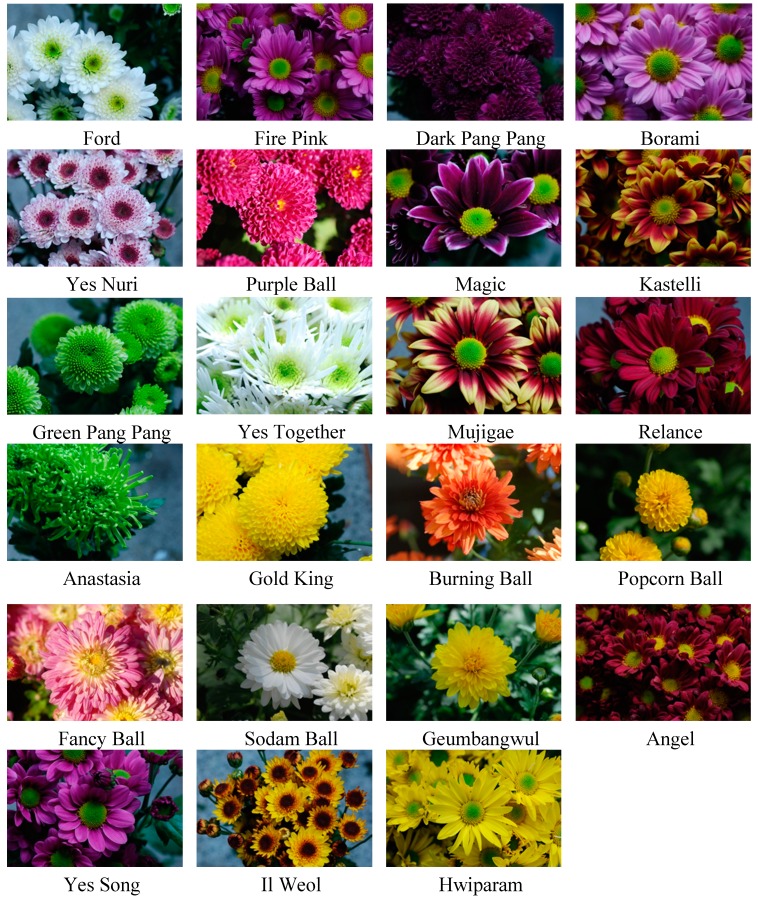
Twenty-three *Dendranthema grandiflorum* Ramat. cultivars.

This suggested that the deep purple and red colored flowers of these cultivars, including “Magic”, “Angel”, “Relance”, and “Green Pang Pang” contained more anthocyanin contents than other flowers (Figure 2).

### 2.2. Analysis of Total Anthocyanin Contents

The cultivars were classified into the following four groups according to the total anthocyanin values: nothing detected (ND), low level (0–5 mg/g Dry Weight (DW)), medium level (5–10 mg/g DW), and high level (>10 mg/g DW) (Table 1). The limit of detection was 0.05 mg/g dry wt. The highest level of total anthocyanin was quantified in flowers of the cultivars “Magic”, followed by “Angel”, “Relance”, and “Green Pang Pang” (Table 3). The cultivars with purple or red colored flowers mostly showed a greater amount of total anthocyanins. On the other hand, the cultivars with medium and low levels of anthocyanin showed light and faint colors or colored spots at the end of petals. The petals of cultivars in the ND group were mostly white, yellow, light green, and very light red in color. This might be because anthocyanin, a strong soluble pigment, mainly affects the development of orange to red and purple to blue colored flowers (Figure 2). 

**Table 3:** Different groups of chrysanthemums according to the amount of total anthocyanin of the twenty-three *Dendranthema grandiflorum* Ramat. cultivars.

ND (0 mg/g dry wt.)	The Low Level (0–5 mg/g dry wt.)	The Medium Level (5–10 mg/g dry wt.)	The High Level (>10 mg/g dry wt.)
Ford	Yes Nuri 0.46 ± 0.28	Dark Pang Pang 6.15 ± 0.11	Green Pang Pang 11.38 ± 0.18
Purple Ball	Il Weol 0.47 ± 0.01	Fire Pink 6.34 ± 0.27	Relance 13.00 ± 0.33
Yes Togethere	Borami 2.08 ± 0.07	Mujigae 7.56 ± 0.18	Angel 13.86 ± 0.17
Anastasiae	Burning Ball 3.37 ± 0.05	Kastelli 7.95 ± 0.04	Magic 18.00 ± 0.84
Gold King	Yes Song 3.63 ± 0.02		
Popcorn Ball	Fancy Ball 4.49 ± 0.07		
Sodam Ball			
Geumbangwul			

The values given in parenthesis under the name of cultivars indicated the mean (mg/g) ± standard deviation (SD) of anthocyanin content of the respective cultivars by triplicate experiments

### 2.3. Analysis of Individual Carotenoid Contents

HPLC analysis of the cultivars suggested that most varieties contained all seven types of carotenoids. In particular, lutein was detected in large amounts, that varied widely among the cultivars. The highest lutein level, which was 26.1 times higher than that in the cultivar with the lowest level (“Fire Pink”) was found in “Il Weol”. The cultivars “Popcorn Ball” and “Anastasia” also had high levels of lutein. The precursor of lutein, α-carotene, was identified in all the varieties. The highest level of α-carotene was found in “Il Weol”, which was 87.50 times higher than that in the cultivar (“Fancy Ball”) with the lowest level. In all the cultivars, lutein content was considerably higher than that of α-carotene, suggesting that α-carotene might be transformed into lutein in large amounts in the cultivars. The second highest carotenoid produced was *trans*-β-carotene. The cultivar “Anastasia” showed the highest accumulation of *trans*-β-carotene, followed by “Green Pang Pang” and “Il Weol”. The cultivar “Fancy Ball” showed the lowest accumulation, which was 39 times lower than that in “Anastasia”. The isomer of β-carotene, 13-*cis*-β-carotene ranged from 0.13 ± 0.01 µg/g DW (“Fancy Ball”) to 5.62 ± 1.75 µg/g DW (“Anastasia”); there was 43.23-fold difference in the concentration of this compound between the two cultivars. Similarly, 9-*cis*-β-carotene, another isomer of β-carotene, showed the highest and lowest levels in “Anastasia’ and “Fancy Ball” respectively, with a 17.86-fold difference between the two.

Previous studies reported that *cis*-isomers are not present in raw carrots, tomatoes, and sweet potatoes, and that 98% of the β-carotene is present as the precursor in raw carrots [15,16]. Our study obtained similar result that all cultivars contained more *trans*-β-carotene contents than 9*-cis*-β-carotene and 13-*cis*-β-carotene. The content of β-cryptoxanthin, which is formed from β-carotene, was 22.89 times more in the cultivar with the highest level (“Il Weol”) than that in the cultivar with the lowest accumulation (“Magic”). Next to lutein and *trans*-β-carotene, the most accumulated carotenoid was zeaxanthin, which is transformed from β-cryptoxanthin. The values among the cultivars ranged from 0.14 ± 0.02 µg/g DW to 4.38 ± 1.26 µg/g DW. The highest level was found in “Il Weol” which was 31.29 times higher than that in the cultivar (“Fire Pink”) with the lowest level. The production of zeaxanthin was higher than that of β-cryptoxanthin in all the cultivars except for “Anastasia” and “Green Pang Pang”, suggesting that β-cryptoxanthin was not completely transformed to zeaxanthin in these two cultivars (Table 4).

**Table 4:** Carotenoid contents of the twenty-three *Dendranthema grandiflorum* Ramat. cultivars.

Cultivars	Carotenoids (μg/g Dry wt.)						
	Lutein	Zeaxanthin	β-Cryptoxanthin	13-*cis*-β-Carotene	α-carotene	Trans-β-carotene	9-*cis*-β-carotene
Ford	52.63 ± 16.68 e–i	0.44 ± 0.10 gh	0.32 ± 0.07 d–g	1.26 ± 0.25 c–e	0.94 ± 0.19 c	15.54 ± 3.21 d	1.46 ± 0.33 de
Fire Pink	11.78 ± 2.53 l	0.14 ± 0.02 h	0.11 ± 0.02 g	0.63 ± 0.08 e–g	0.15 ± 0.03 ef	6.07 ± 1.17 f–h	0.55 ± 0.11 fg
Dark Pang Pang	15.13 ± 3.16 kl	0.15 ± 0.02 h	0.13 ± 0.04 g	0.42 ± 0.03 fg	0.28 ± 0.04 d–f	4.24 ± 0.61 f–h	0.37 ± 0.07 g
Borami	37.75 ± 2.31 g–l	0.37 ± 0.02 gh	0.13 ± 0.02 g	0.67 ± 0.10 e–g	0.29 ± 0.05 d–f	5.92 ± 0.39 f–h	0.60 ± 0.03 fg
Yes Nuri	39.70 ± 2.26 g–l	0.19 ± 0.01 h	0.14 ± 0.02 g	1.00 ± 0.11 d–f	0.66 ± 0.19 c–e	12.56 ± 0.69 de	1.13 ± 0.08 ef
Purple Ball	29.60 ± 3.04 h–l	0.26 ± 0.01 h	0.10 ± 0.01 g	1.12 ± 0.16 c–f	0.13 ± 0.01 ef	10.27 ± 1.22 d–f	0.84 ± 0.08 e–g
Magic	17.84 ± 1.21 j–l	0.42 ± 0.02 gh	0.09 ± 0.01 g	0.73 ± 0.05 e–g	0.24 ± 0.02 d–f	6.09 ± 0.34 f–h	0.53 ± 0.04 fg
Kastelli	89.46 ± 6.93 cd	2.64 ± 0.17 bc	0.54 ± 0.06 c–e	1.16 ± 0.07 c–f	0.51 ± 0.06 c–f	7.13 ± 0.60 e–h	0.84 ± 0.05 e–g
Green Pang Pang	59.51 ± 3.83 d–h	0.31 ± 0.01 gh	0.67 ± 0.04 c	2.81 ± 0.15 b	1.98 ± 0.14 b	30.63 ± 1.13 b	2.74 ± 0.13 c
Yes Together	34.03 ± 2.28 h–l	1.06 ± 0.05 ef	0.26 ± 0.01 e–g	0.57 ± 0.03 e–g	0.56 ± 0.01 c–f	5.01 ± 0.06 f–h	0.53 ± 0.01 fg
Mujigae	47.49 ± 5.70 f–k	0.95 ± 0.11 f	0.58 ± 0.10 cd	1.19 ± 0.12 c–f	0.18 ± 0.08 ef	9.00 ± 1.21 e–g	0.79 ± 0.10 fg
Relance	24.55 ± 9.96 i–l	1.67 ± 0.07 d	0.48 ± 0.11 c–f	0.52 ± 0.03 e–g	0.18 ± 0.01 ef	3.05 ± 0.11 gh	0.36 ± 0.01 g
Anastasia	98.88 ± 20.46 c	0.33 ± 0.04 gh	1.85 ± 0.34 a	5.62 ± 1.75 a	3.32 ± 0.85 a	55.77 ± 14.34 a	5.18 ± 1.42 a
Gold King	33.58 ± 4.29 h–l	2.78 ± 0.12 b	0.62 ± 0.30 cd	0.76 ± 0.15 e–g	0.24 ± 0.04 d–f	4.09 ± 0.63 f–h	0.46 ± 0.06 fg
Burning Ball	68.96 ± 12.82 c–g	1.53 ± 0.39 de	0.32 ± 0.04 d–g	0.86 ± 0.12 e–g	0.23 ± 0.05 d–f	6.29 ± 1.16 e–h	0.96 ± 0.11 e–g
Popcorn Ball	165.48 ± 32.75 b	2.59 ± 0.22 bc	0.67 ± 0.16 c	1.79 ± 0.36 cd	1.88 ± 0.40 b	15.17 ± 2.26 d	1.98 ± 0.32 d
Fancy Ball	19.71 ± 0.78 i–l	0.30 ± 0.02 gh	0.13 ± 0.09 g	0.13 ± 0.01 g	0.04 ± 0.01 f	1.43 ± 0.08 h	0.29 ± 0.02 g
Sodam Ball	31.21 ± 1.62 h–l	0.82 ± 0.03 fg	0.11 ± 0.01 g	1.21 ± 0.16 c–f	0.12 ± 0.02 ef	10.50 ± 0.52 d–f	0.88 ± 0.07 e–g
Geumbangwul	83.06 ± 7.77 c–e	2.80 ± 0.15 b	1.31 ± 0.18 b	1.11 ± 0.23 c–f	0.17 ± 0.00 ef	8.49 ± 1.29 e–g	0.89 ± 0.11 e–g
Angel	27.84 ± 8.15 h–l	2.89 ± 0.12 b	0.32 ± 0.04 d–g	0.69 ± 0.06 e–g	0.24 ± 0.02 d–f	2.94 ± 0.16 gh	0.40 ± 0.02 g
Yes Song	73.34 ± 1.11 c–f	1.19 ± 0.03 d–f	0.19 ± 0.02 fg	1.89 ± 0.10 c	0.41 ± 0.03 c–f	16.46 ± 1.17 cd	1.49 ± 0.11 de
Il Weol	307.22 ± 64.98 a	4.38 ± 1.26 a	2.06 ± 0.41 a	2.93 ± 0.73 b	3.50 ± 0.98 a	21.92 ± 5.37 c	3.55 ± 0.87 b
Hwiparam	49.76 ± 6.39 f–j	2.20 ± 0.25 c	0.52 ± 0.29 c–e	1.06 ± 0.12 d–f	0.77 ± 0.08 cd	4.55 ± 0.66 f–h	0.94 ± 0.13 e–g

All values are written as the mean (μg/g) ± standard deviation (SD) of three replications. The amounts are expressed as μg of the target compound per g of the plant on a dry weight basis. Means values in a common letter were not significantly different at *p <* 0.05 using Duncan Multiple Range Test. The background color in each column presents the flower color of the cultivar.

### 2.4. Analysis of Total Carotenoid Contents

Analysis of the petals of the chrysanthemum varieties revealed the presence of carotenoid components such as lutein, zeaxanthin, β-cryptoxanthin, α-carotene, 13-*cis*-β-carotene, *trans*-β-carotene, and 9-*cis*-β-carotene, and the concentration of these components varied significantly (Table 4). The cultivars were classified on the basis of the level of total carotenoid production (Table 5). 

**Table 5:** Different groups of chrysanthemum*s* according to the amount of total carotenoid in the twenty-three *Dendranthema grandiflorum* Ramat. cultivars.

The Low Level	The Medium Level	The High Level
(0–50 μg/g Dry wt.)	(50–100 μg/g Dry wt.)	(>100 μg/g Dry wt.)
Fire Pink 19.43 ± 3.94	Yes Nuri 55.38 ± 3.26	Kastelli 102.28 ± 7.70
Dark Pang Pang 20.72 ± 3.94	Hwiparam 59.80 ± 7.23	Anastasia 170.95 ± 37.10
Fancy ball 22.03 ± 0.87	Mujigae 60.19 ± 6.77	Popcorn Ball 189.57 ± 36.28
Magic 25.94 ± 1.64	Ford 72.59 ± 20.80	Il Weol 345.56 ± 74.29
Relance 30.81 ± 9.80	Burning Ball 79.15 ± 14.29	
Angel 35.31 ± 8.00	Yes Song 94.97 ± 0.53	
Yes Together 42.02 ± 2.30	Geumbangwul 97.83 ± 9.16	
Purple Ball 42.33 ± 4.49	Green Pang Pang 98.66 ± 5.03	
Gold King 42.54 ± 5.37		
Sodam Ball 44.85 ± 2.33		
Borami 45.74 ± 2.80		

The values given in parenthesis under the name of cultivars indicated mean (μg/g) ± standard deviation (SD) of carotenoid content of the respective cultivars by triplicate experiments.

The cultivars with total carotenoid contents ranging from 0 to 50 µg/g DW and 50 to 100 µg/g DW were grouped as low level and medium level, respectively, and those with contents of more than 100 µg/g DW were grouped as high level. The highest level was quantified in the cultivar “Il Weol”, followed by “Popcorn Ball”, “Anastasia” and “Kastelli”. The lowest level was recorded for the cultivar “Fire pink” (Table 5). The low and medium level cultivars usually had faintly bluish, purplish, whitish, or reddish colored flowers. On the other hand, high level cultivars generally showed deep yellow or green colored petals. Interestingly, cultivar “Kastelli” had red colored petals. This cultivar might accumulate reddish carotenoids that are absent in yellow petals, since a previous study reported that *Adonis aestivalis* excessively synthesized the red carotenoid astaxanthin, resulting in blood-red colored petals [17]. Anthocyanin analysis revealed that the cultivar “Magic” was the most suitable source of C3g and C3mg since it accumulated the largest amount of these anthocyanins. The cultivar “Relance’ showed the highest accumulation of the unknown anthocyanin (Table 2). According to carotenoid analysis, the cultivar “Il Weol”, which had deep yellow petals, showed the highest accumulation of carotenoids, including lutein, and zeaxanthin. *trans*-β-Carotene, 9-*cis*-β-carotene, and 13-*cis*-β-carotene were quantified in the largest amounts in the cultivar “Anastasia”, which has green colored petals. Additionally, the highest accumulation of β-cryptoxanthin and α-carotene was noted in the cultivars “Anastasia” and “Il Weol” (Table 4). 

Comparison of anthocyanin and carotenoid analyses confirmed the finding revealed by Alkema, *et al.* [18] that varying petals colors are obtained due to the presence and interaction of different carotenoids and flavonoids. Most yellow flowers result from the presence of carotenoids (especially, xanthophylls), whereas anthocyanins are responsible for most red, blue, and purple colored petals. The cultivars “Magic”, “Angel”, and “Relance”, which have redder and more purplish colored petals (Figure 2), were grouped into high level group based on the high amount of total and individual anthocyanins (Table 3). On the other hand, according to carotenoid contents, they were classified into the low level group (Table 5). The cultivars “Il Weol”, “Popcorn Ball”, and “Anastasia” with deep yellow and green colored petals (Figure 2) showed the highest levels of carotenoid contents (Table 5). However, according to anthocyanin contents, “Popcorn Ball” and “Anastasia” were included in the ND group, and “Il Weol” was included in the low level group. Other cultivars also showed similar findings except for “Green Pang Pang” and “Kastelli”.

Interestingly, “Green Pang Pang” which had a high level of anthocyanins (11.38 ± 0.18 mg/g DW) as well as a medium level of carotenoids (98.66 ± 5.03 μg/g DW) had deep green colored petals (Table 3 and Table 5). Even if it had high level of anthocyanins, it did not have the typical orange and red to purple color range in its petals. It was therefore assumed that the combination of the pigments influenced the color determination of petals. Similarly, “Kastelli”, which had a high level of carotenoids (102.28 ± 7.70 μg/g DW) and a medium level of anthocyanins (7.95 ± 0.04 mg/g DW), showed orange and red colored petals (Table 3 and Table 5). The flower color is known to be determined on the basis of the production, interaction, and breakdown of pigments such as carotenoids located in plastids and anthocyanins found in vacuoles [19]. In particular, orange colored petals result from the coexistence of both those pigments. The ratio of the amount of these pigments allows the variation in orange color in the petals of *Psorophora howardii* [20].

Most cultivars that were classified as ND or low level showed faint/pale colored or white colored petals. Although these cultivars lack pigments, they might have factors that inhibit the pigments. It was reported that the biosynthesis of carotenoids could impart white colors to petals in chrysanthemums, since the component was subsequently degraded to colorless compounds by a factor, carotenoid cleavage dioxygenase (CmCCD4a) [21].

## 3. Experimental Section

### 3.1. Plant Materials

Twenty-three cultivars of chrysanthemum (*Dendranthema grandiflorum* Ramat.) in the study were “Ford”, “Fire Pink”, “Dark Pang Pang”, “Borami”, “Yes Nuri”, “Purple Ball”, “Magic”, “Kastelli”, “Green Pang Pang”, “Yes Together”, “Mujigae”, “Relance”, “Anastasia”, “Gold King”, “Burning Ball”, “Popcorn Ball”, “Fancy Ball”, “Sodam Ball”, “Geumbangwul”, “Angel”, “Yes Song”, “Il Weol”, and “Hwiparam” (Figure 1). The cultivars were propagated through root ball division from mother plants. The divided roots were grown in soil mixture with sand on the surface at Yesan Chrysanthemum Experiment Station, Yesan-gun, Chungcheongnam-do, Korea. The plants were grown for 4 months until flowering and flowers were sampled for anthocyanin and carotenoid analysis. 

### 3.2. Extraction of Anthocyanins, Including C3g and C3mg, and HPLC Analysis

The flowers of the twenty-three cultivars were individually ground to powder after freeze-drying. Next, the powder (0.1 g) was added to a 2.0 mL Eppendorf tube, and then extraction solution (water-formic acid, 95:5 (*v*/*v*), 2 mL) was added to the tube. The tube was vortexed and sonicated for 5 min. Subsequently, the tube was centrifuged at 12,000 rpm at 4 °C for 15 min using a MICRO 17R micro-centrifuge (Hanil, Incheon, Korea). The supernatant was filtered through a 0.45-µm polytetrafluoro-ethylene hydrophilic syringe filter and collected into vials. Each extraction was repeated in triplicate. C3gl, C3gm, and unknown anthocyanin contents were separated using a reversed-phase Synergi 4-µm POLAR-RP 80A (250 × 4.6 mm; particle size, 4 µm; Phenomenex, Torrance, CA, USA) equipped with a Security Guard Cartridge kit (AQ C18; 4 × 3.0 mm i.d.; KJO-4282; Phenomenex) using a 1200 series HPLC system (Agilent Technologies, Palo Alto, CA, USA); the oven temperature was set to 40 °C; detection wavelength, 520 nm; and flow rate, 1.0 mL/min. Solvent A was water-formic acid, 95:5 (*v*/*v*), and acetonitrile-formic acid, 95:5 (*v*/*v*) was used as solvent B in the mobile phase. The gradient conditions were set as follows: a linear step from 5% solvent B for 0.0 min, 50% solvent B for the next 30.00 min, followed by a rapid drop to 5% B for 30.10 min, and then isocratic conditions with 5% B for 40.00 min (total 100.10 min). Individual C3gl and C3gm levels were quantified using their HPLC area and response factor; they were compared with an external standard, *i.e.*, 5 mL of sinigrin solution (0.1 mg/mL) that was subjected to the same extraction process [19].

### 3.3. LC/ESI-MS Analysis for the Quantification of Anthocyanin Contents

The ESI-MS data were analyzed using a 4000 Qtrap LC/MS/MS system (Applied Biosystems Instrument, Foster City, CA, USA) in the positive ion mode ([M + H]^+^) connected with an Agilent 1200 series HPLC. The LC/MS analytical conditions for anthocyanins in the chrysanthemum cultivars were set as follows: scan range, 100–1300 m/z; scan time, 4.80 s; curtain gas, 20.00 psi (N_2_); heating gas temperature, 550 °C ; nebulizing gas, 50.00 psi; heating gas, 50.00 psi; ion spray voltage, 5500 V; declustering potential, 100 V; and entrance potential, 10 V.

### 3.4. Extraction and HPLC Analysis of Carotenoids

The carotenoids were extracted and measured by HPLC as described previously by our group [22]. Eight carotenoids standards (lutein, zeaxanthin, β-cryptoxanthin, 13*Z*-β-carotene, α-carotene, *trans*-β-carotene, 9*Z*-β-carotene, and β-*apo*-8ʹ-carotenal) were obtained from CaroteNature (Lupsingen, Switzerland). Heat and saponification are required to release carotenoids from the samples and remove oils interfering with chromatographic analysis. Briefly, carotenoids were released from the flower samples (0.05 g) by adding ethanol (3 mL) containing 0.1% ascorbic acid (*w*/*v*), vortex mixing for 20 s and placing in a water bath at 85 °C for 5 min. The carotenoid extracts were saponified with potassium hydroxide (120 µL, 80% *w*/*v*) at 85 °C for 10 min. After saponification, the samples were placed on ice, and cold deionized water (1.5 mL) was added. Based on its carotenoid characteristics and retention time, β-*apo*-8ʹ-carotenal (0.2 mL, 25 µg/mL) was chosen as an internal standard. To separate the layers, the carotenoids were extracted twice with hexane (1.5 mL) by centrifugation at 1200× *g* for 5 min at 4 °C. The aliquots of the extracts were then dried under a stream of nitrogen and redissolved in 50:50 (*v*/*v*) dichloromethane/methanol before the HPLC analysis. Each extraction was repeated in triplicate. The carotenoids were separated in a C30 YMC column (250 × 4.6 mm, 3 µm; YMC Co., Kyoto, Japan) by an Agilent 1100 HPLC instrument (Massy, France) equipped with a photodiode array detector and the chromatograms were generated at 450 nm. Solvent A consisted of methanol/water (92:8 *v*/*v*) with 10 mm ammonium acetate and solvent B consisted of 100% methyl *tert*-butyl ether. The gradient elution was performed at 1 mL/min under the following conditions (A%/B%): 0 min, 90/10; 20 min; 83/17; 29 min, 75/25; 35 min, 30/70; 40 min, 30/70; 42 min, 25/75; 45 min, 90/10; and 55 min, 90/10. Identification of carotenoids was carried out by HPLC through the combined use of the retention time and co-elution with available authentic standards. For quantification, calibration curves were drawn by plotting 4 different concentrations of carotenoid standards, according to the peak area ratios with β-*apo*-8ʹ-carotenal. The linear equations for lutein, zeaxanthin, β-cryptoxanthin, 13*Z*-β-carotene, α-carotene, *trans-*β-carotene, and 9*Z*-β-carotene were y = 0.1847x + 0.1214, y = 0.4712x − 0.0284, y = 0.3329x + 0.0338, y = 0.3361x – 0.0220, y = 0.5479x – 0.0805, y = 0.2154x + 0.1814, and y = 0.4284x + 0.0339, respectively. The calibration curves of seven carotenoids measured at different ranges (1–5 μg/mL) were linear with good correlation coefficients (*r*^2^ = 0.954–0.999).

### 3.5. Statistical Analysis

Data was analyzed using the computer software Statistical Analysis System (SAS, system 9.3, 2012; SAS Institute, Inc., Cary, NC, USA). Means were separated by Duncan’s Multiple Range Test (DMRT). The experimental results were presented as mean ± standard deviation of triplicate experiments.

## 4. Conclusions

In this study, the pigments such as anthocyanins and carotenoids were profiled and quantified in twenty-three cultivars of chrysanthemum (*Dendranthema grandiflorum* Ramat.). The results of our study suggested the cultivars “Magic”, “Angel”, and “Relance”, which had deep red or purple colored petals, are suitable powerful sources of anthocyanins due to their high anthocyanin contents, whereas “Il Weol”, “Popcorn Ball”, and “Anastasia”, which showed deep yellow or green colored petals, areconsidered a good source for the carotenoids because of high production of carotenoid contents. Interestingly, “Green Pang Pang” showed deep green colored petals even though it contained a high level of anthocyanins and a medium level of carotenoids. Similarly, “Kastelli” with high levels of carotenoids and a medium level of anthocyanins showed orange and red colored petals. Consequently, it was confirmed that the pigments responsible for the petals colors are found in most cultivars and their pigment components affect the flower colors of the cultivars. Further researches on the molecular characterization and the metabolic profiling are necessary to explain the accumulation and interaction of the secondary metabolites leading to the various petals colors in the cultivars.
